# Comparison of Statistical Tests for Association between Rare Variants and Binary Traits

**DOI:** 10.1371/journal.pone.0042530

**Published:** 2012-08-09

**Authors:** Silviu-Alin Bacanu, Matthew R. Nelson, John C. Whittaker

**Affiliations:** 1 Quantitative Sciences, GlaxoSmithKline, Research Triangle Park, North Carolina, United States of America; 2 Virginia Commonwealth University, Richmond, Virginia, United States of America; 3 Quantitative Sciences, GlaxoSmithKline, Stevenage, United Kingdom; Johns Hopkins University, United States of America

## Abstract

Genome-wide association studies have found thousands of common genetic variants associated with a wide variety of diseases and other complex traits. However, a large portion of the predicted genetic contribution to many traits remains unknown. One plausible explanation is that some of the missing variation is due to the effects of rare variants. Nonetheless, the statistical analysis of rare variants is challenging. A commonly used method is to contrast, within the same region (gene), the frequency of minor alleles at rare variants between cases and controls. However, this strategy is most useful under the assumption that the tested variants have similar effects. We previously proposed a method that can accommodate heterogeneous effects in the analysis of quantitative traits. Here we extend this method to include binary traits that can accommodate covariates. We use simulations for a variety of causal and covariate impact scenarios to compare the performance of the proposed method to standard logistic regression, C-alpha, SKAT, and EREC. We found that i) logistic regression methods perform well when the heterogeneity of the effects is not extreme and ii) SKAT and EREC have good performance under all tested scenarios but they can be computationally intensive. Consequently, it would be more computationally desirable to use a two-step strategy by (i) selecting promising genes by faster methods and ii) analyzing selected genes using SKAT/EREC. To select promising genes one can use (1) regression methods when effect heterogeneity is assumed to be low and the covariates explain a non-negligible part of trait variability, (2) C-alpha when heterogeneity is assumed to be large and covariates explain a small fraction of trait’s variability and (3) the proposed trend and heterogeneity test when the heterogeneity is assumed to be non-trivial and the covariates explain a large fraction of trait variability.

## Introduction

Genome-wide association studies (GWAS) have found many genetic variants associated with a wide range of traits [1]–[4]. There is evidence that for most traits, common variants identified in GWAS collectively explain a smaller fraction of phenotypic variability than expected [2], [5], [6]. Consequently, a considerable portion of the genetic contribution to phenotypic variability remains unknown [7]. One possible explanation is that rare genetic variation, which is poorly assayed or tagged by current GWAS platforms, may account for much of that missing variation [8]. There are a growing number of examples of rare variants having large effects on complex traits [9]–[11].

Advances in short-read sequencing technology have made the investigation of low frequency variants increasingly cost-effective. In turn, the availability of large-scale sequencing studies are spurring the development of statistical methodology for their analysis. The first wave of methods applied to the analysis of rare variation collapsed the genotypes for all rare variants (RVs) from a defined genetic unit, e.g. a gene, into a single carriage status variable. Subsequently, the frequency of RV carriers was contrasted between cases and controls (or extreme tails of a quantitative trait distribution) [12]. The collapsing method can also be performed in a regression setting by treating the binary trait as a dependent variable and regress it on carriage status or on carriage status and covariates [13]. While these methods can accommodate covariates, due to collapsing they lose power when RV effects are heterogeneous. This is a concern since, we know that the effects of RVs in many genes can be heterogeneous, e.g. *PCSK9* [14], [15] and *CASR* [16].

**Table 1:** Simulation design parameters.

Parameter Name	Parameter	Design levels
Sample size	*n*	1000 cases and 1000 controls
Prevalence	*K*	0.1
Fraction of trait’s variability explained by covariate (%)	*Rsq*	[14]
True damage class	*D_t_*	[30]
Effect size (SDs)	*δ*	0 to 1 in steps of 0.05
Heterogeneity parameter	*ζ*	{0.5, 0.8, 1}
Percentiles coding sequence lengths (coding base pairs)	*CDS*	{10, 50, 90} ({361,1209, 4057})
Number simulations at each design level	*m*	250 for the empirical power (*δ>0*) 25,000 for the size of the test (*δ = 0*)

**Figure 1:**
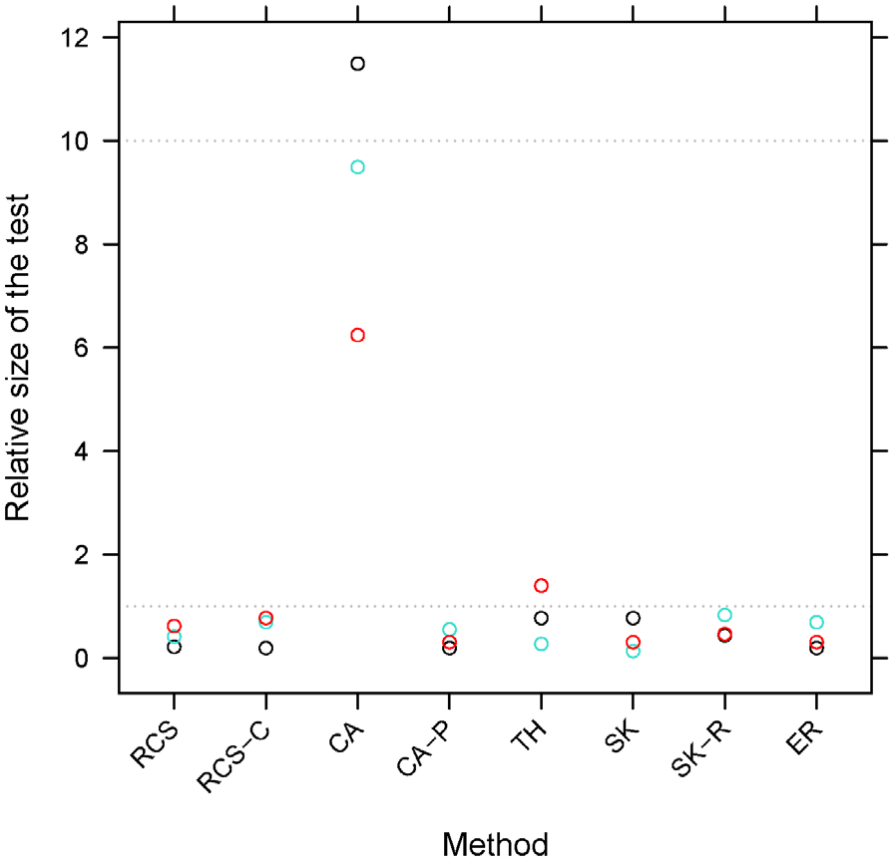
Relative size of the test* for 1000 cases and 1000 controls at a type I error of 10^−3^. The size of the test estimated empirically from 25,000 simulations. Black, turquoise and red circles correspond to gene CDS equal to 10, 50, and 90 percentiles of the human gene CDS distribution, respectively. Methods: RCS – (logistic) regression on carriage status, RCS-C – (logistic) regression on carriage status and covariates, CA – C-alpha test, CA-P – C-alpha test with permutations, TH – test of trend and heterogeneity, SK – SKAT, SK-R – SKAT with (parametric bootstrap) resampling, ER – EREC. *The ratio of the size of the test to the nominal type I error rate.

The possibility of effect heterogeneity led researchers to develop methods that can accommodate such a scenario. We previously developed methods for quantitative traits which allow for the heterogeneity of RV effects [17]. To accommodate the fact that RVs in the same gene can result in increased or decreased phenotypic values, our method tests if RV carriers have increased square deviations of phenotype from its mean. One of the first methods to test for association between binary traits and RVs in the presence of both risk and protective variants was the weighted sum approach of Ionita-Laza et al. [18]. A somewhat similar approach is the C-alpha test that, similar to the above weighted sum approach, accommodates heterogeneity but does not accommodate covariates [19]. C-alpha is a test of extra-binomial variance in the proportion of cases within each variant. Subsequently, kernel based adaptive clustering (KBAC) was proposed to accommodate both heterogeneity and covariates [20]. A similarity regression approach was proposed to jointly analyze common and rare variants in the presence of heterogeneous effects [21]. Lately, there were also proposals for methods which can test a wide range of statistical models in the presence of covariates and effect heterogeneity. Due to the wide range of models, these methods generalize many previous approaches. One of the first of these general methods was the Sequence Kernel Association Test (SKAT) [22]. SKAT uses a kernel regression machine to model the genotype phenotype association. A related general approach is the Estimated REgression Coefficients (EREC) method [23]. EREC uses a general linear model framework to generalize most RV tests.

**Figure 2:**
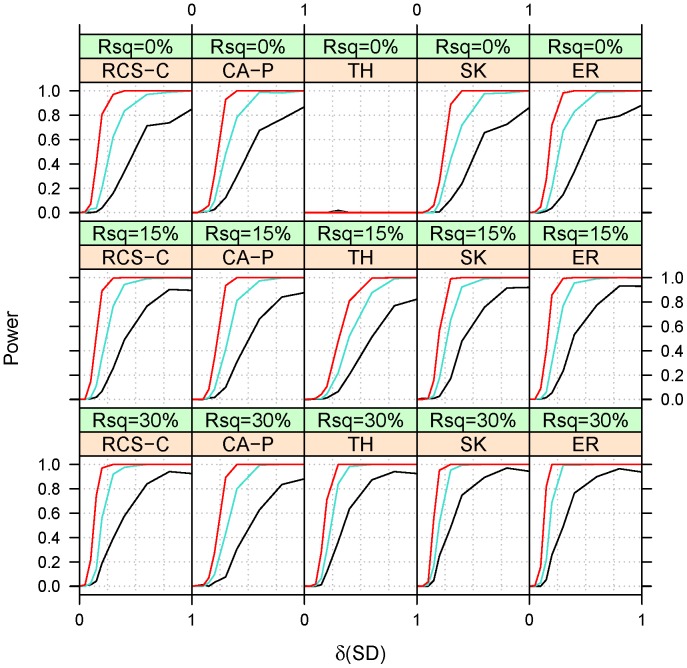
Empirical power at a type I error of 10^−3^ for Scenario 1 under homogeneity (ξ = 1). The power estimated from 250 simulations. The covariate is assumed to be explaining a fraction (Rsq) equal to 0, 10 or 20% of the variability in binary trait. Power is presented for 10% (black), 50% (turquoise) and 90% (red) percentiles of CDS length. See Fig. 1 for background and abbreviation.

We extend the methods we previously developed for quantitative traits to the analysis of binary traits in the presence of covariates. The performance of the proposed and competing methods is evaluated by a simulation design with varying heterogeneity levels and covariate influence. Based on the results we make recommendations on how to choose the most desirable method (or combination of methods) based on the influence of covariates and the potential level of heterogeneity of RV effects.

**Figure 3:**
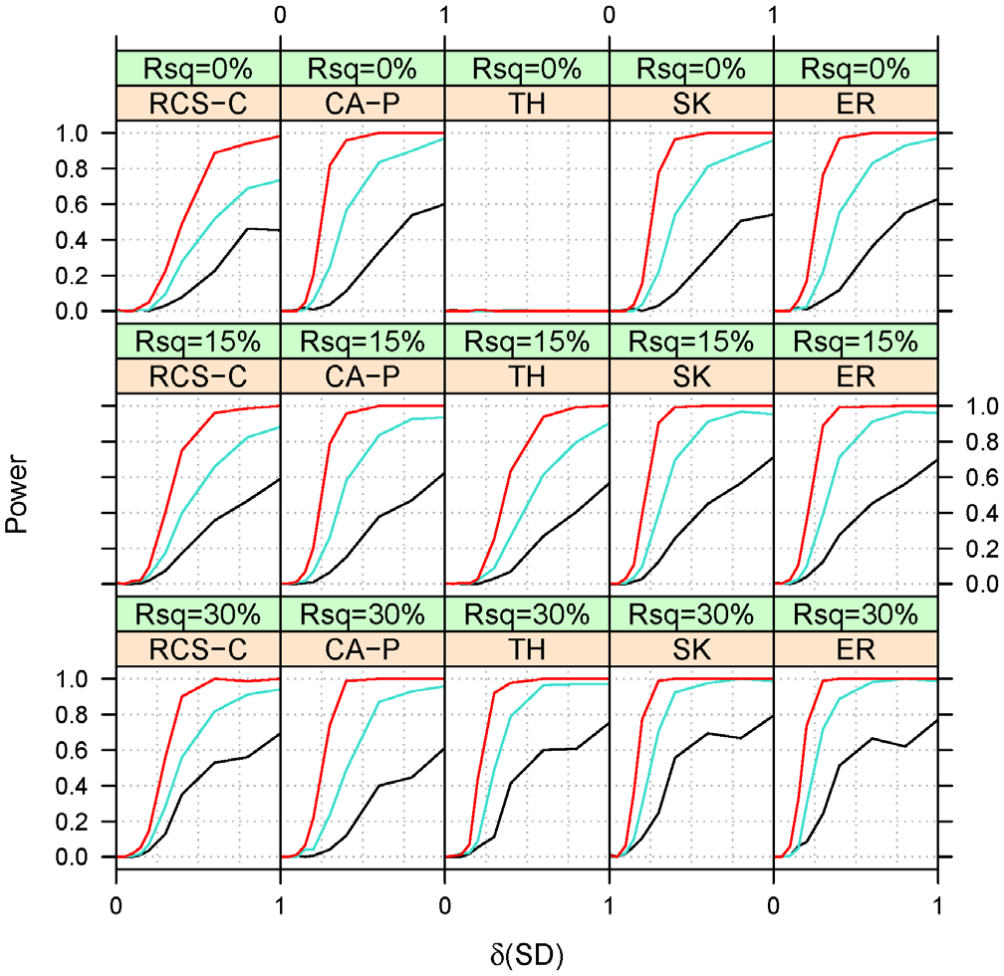
Empirical power at a type I error of 10^−3^ for Scenario 1 under heterogeneity (ξ = 0.5). See Fig. 1 and 2 for background and abbreviation.

**Figure 4:**
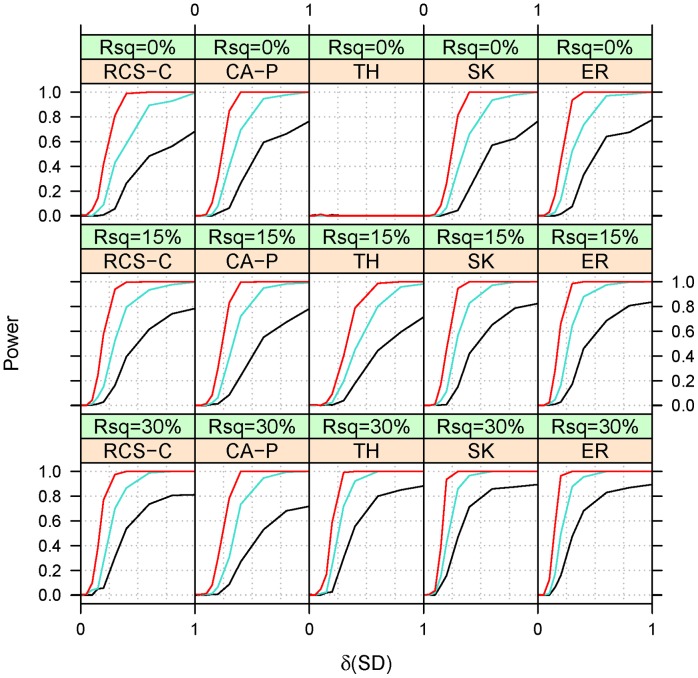
Empirical power at a type I error of 10 for Scenario 1 under partial heterogeneity (ξ = 0.8). See Fig. 1 and 2 for background and abbreviation.

## Methods

We previously developed a trend and heterogeneity (TH) test for quantitative traits which accommodates heterogeneity [17]. Such a test was developed because in practice the true causal model realistically lies somewhere between homogeneity and complete heterogeneity (the effect of the variant is equally likely to be positive or negative). To develop a powerful test for such circumstances, we start from the second moment equality: 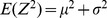, where 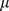 is the mean and *σ^2^* is the variance of random variable 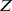. Therefore, the square of quantitative trait, 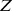, naturally incorporates information about both the trend (i.e. the mean), and the heterogeneity (i.e. the variance). Thus, assuming 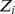, *i* = 1,…*m*, are the quantitative phenotypes of RV carriers, we proposed the trend and heterogeneity statistic: 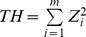. The statistical significance of TH is computed very fast empirically by resampling 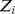 of the *m* RV carriers from the larger set of quantitative values for the entire sample.

TH can be adapted to binary traits in a straightforward manner by: (1) using a logistic regression of the binary trait on the covariates to obtain Pearson’s residuals and (2) treating Pearson’s residuals as a quantitative trait in a TH test (see [24] for a similar treatment). However, by not analyzing covariates and carriage status simultaneously, the straightforward TH adaption does not fully use the information available.

To assess the performance of the proposed method we compare its performance to the performance of several alternatives. In this study we include simple regression, C-alpha (CA), SKAT and EREC methods. The regression methods use a logistic regression of the binary trait on carriage status (RCS) or on carriage status and covariates (RCS-C). For CA (our implementation based on code from Dr. Kathryn Roeder), we present the size of the test based on i) asymptotic p-values (CA) and ii) permutation p-values (CA-P). SKAT (version 0.72) performance was assessed at the default settings (i.e. linear kernel, etc.) using i) asymptotic p-values (SK) and ii) p-values derived from parametric bootstrap resampling (SK-R). EREC (SCORE-Seq version 2.0) performance was assessed at the default settings with the exception of the minimum allele count. Because many variants had only a few minor alleles and we wanted to include all variants in our analyses, we set EREC’s minor allele count parameter to zero. EREC statistical significance is assessed adaptively from up to one million permutations. While KBAC should be able to accommodate heterogeneity and covariates, the software implementing the method was not available at the time we carried out our initial simulations. Consequently, we did not include KBAC in this study.

We use a simulation design (Table 1) to compare the performance of the above mentioned methods when they are used for a gene level analysis of non-synonymous RVs. We assume a sample of 1000 cases and 1000 controls for a binary trait with a prevalence K = 10%. A potentially relevant covariate was assumed to explain a fraction (Rsq) equal to 0%, 15%, or 30% of binary trait variability. We simulate data sets by (1) assuming that the probability of carrying an RV is 1% per 500 bp of coding sequence (i.e. the larger genes contain more RVs) [17], [25] (2) simulating a latent and normally distributed variable based on the RVs in each subject (see [17]), (3) using the latent variable in a threshold model to generate a binary trait with prevalence K and (4) sampling the required number of cases and controls.

Similar to our previous work [17], we define RVs as having a minor allele frequency less than 0.5%. Since the expected number of rare alleles per subject depends on the length of the coding sequence (CDS), in each simulation we generate variant sites (SNPs) having their frequencies independently drawn from a Wright’s distribution [18], [26] until their cumulative minor allele frequency is closest to the expected probability of carrying a rare allele in the gene under investigation (i.e. 1% for each 500 CDS bps [17]). For Wright’s distribution, 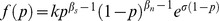 (where *p* is the mutation allele frequency), 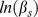 *is* assumed to be uniform between 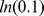 and 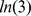, 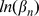 uniform between 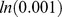 and 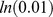 and *σ* is assumed to be *0* with probability *0.5* and distributed as a uniform between *0* and *20* with probability *0.5* [26]. Large scale sequencing studies show that the Wright formula with our choice of parameters underestimates the occurrence of very rare variants in human populations [27]. However, there is not a substantial difference in frequencies of rarer variants between our simulations and applied sequencing studies. Consequently, the conclusions derived from our simulation design are likely to be very similar to those derived from a design based on real sequencing data.

Let 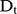 be the (true) deleteriousness class of RVs, i.e. the deleterious class of the RV a subject carries in a gene and zero otherwise. (For the unlikely case of multiple RVs in the same gene and subject, we retain only the most deleterious variant.) 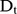 is assumed to be a numerical variable with integer levels of 0 (for subjects not possessing RVs) to 3 (very deleterious). For each variant, 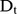 is sampled from 0 to 3, with the probability vector (0.26,0.16,0.36,0.22), as estimated from population genetics studies [28] (see Table 4 of Boyko et al. [28] or Table S1 in Supplementary Material). To facilitate the investigation of various heterogeneity models, each variant was also assigned a sign, 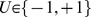, for its effect on trait, i.e. each variant was simulated to be either risk increasing (+1) with probability 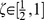 or risk decreasing (−1) with probability 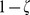. 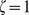 corresponds to homogeneity, 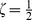 corresponds to complete heterogeneity and intermediate values of 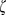 correspond to varying levels of partial heterogeneity. We note that both 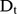 and 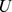
are variant specific, i.e. they have the same value for all carriers of the variant. Let *G* be the variable denoting RV carriage status, i.e. the indicator of the deleterious class being nonzero − I[*D_t_*>0]. With these assumptions we model the latent trait, *Z*, as follows:


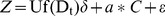


where


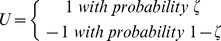


is the sign of the effect for the rare variant, 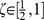 is the heterogeneity parameter controlling the relative frequency of mutations increasing the trait levels, 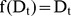 for linear (Scenario 1) model in which the true damage class reflects effect size and 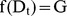 for homogeneity of the effect magnitude model (Scenario 2). 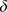 is the difference in phenotype means between two adjacent levels of the explanatory variable (in standard deviations). The parameter 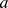 is the coefficient of the covariate, 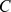, which explains 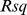 of the binary trait heritability. The error term, 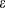, is assumed to be normally distributed. A subject with a latent variable, 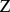, is assigned to be a i) case if 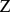 is above the threshold defined by the 1−*K* percentile (i.e. 90% for our choice of prevalence) of the latent trait distribution and ii) control if 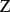 is below this threshold.

For every gene level analysis, the number of rare variants, and therefore power, depends on the length of CDS. Consequently, the power and size of the test for each method was estimating assuming CDS are equal to the {*10, 50, 90*} percentile (i.e. {*361,1209, 4057*} coding base pairs) of the CDS for human genes as estimated [17] from RefSeq [29].

## Results

Under the null hypothesis of no association between trait and RVs, all methods with the exception of asymptotic distribution CA control the type I error (Fig. 1). Methods that control the type I error show a slight tendency to be more conservative at short gene lengths, likely due to the discreteness of the distribution. Thus, for a fair comparison between methods, we estimate the power of CA, TH, SKAT and EREC under the alternative hypothesis by using permutation (resampling for SKAT) tests. Because they need to recompute the statistics for each permutation, the running time for permutation based SKAT and EREC inference was almost two orders of magnitude larger than the running time of TH and CA-P.

The power was assessed under three heterogeneity settings: homogeneity (ξ = 1, Fig. 2), heterogeneity (ξ = 0.5, Fig. 3) and partial heterogeneity (ξ = 0.8, Fig. 4). The qualitative features of power under the two scenarios (linear and magnitude homogeneity) are quite similar, which suggests that collapsing in a regression framework is actually relatively robust to heterogeneity in the magnitude of effects. Due to this similarity, we present only the power under the scenario of effect linearity. Because RCS-C has equal power to RCS when the covariate explains 0% (i.e. Rsq = 0%) of the trait and it is greater than RCS when Rsq is 15% or greater, we omit RSC from the presentation of power estimates.

As expected, RCS-C has the greatest power under homogeneity (ξ = 1), except probably at the shorter gene lengths where SK and ER perform better. The different behavior of RCS-C at the shorter gene lengths is due its greater conservativeness at these lengths. While the advantage of RCS-C over CA-P grows with the increase in the proportion of the trait variance explained by the covariate, its advantage over TH, SK and ER decreases with the increase in the proportion of the trait variance explained by the covariate.As expected, the power of TH is close to zero when Rsq = 0%. This low power is the direct result of the almost identical residuals obtained from regressing the binary trait on an uncorrelated covariate. The power of RCS-C, SK and ER increases with an increase in Rsq. This is a result of the decreased mean square error of the genetic coefficients induced by the increased fraction explained by the biologically relevant covariate.

Under heterogeneity (ξ = 0.5), CA-P performs best when Rsq = 0%, i.e. when the covariate does not explain any fraction of the variability in the binary trait. However, SK and ER performs almost as well at Rsq = 0% and performs best for other values of Rsq. At higher Rsq, TH performs better than CA-P and almost as well as SK and ER. SK and ER have a very similar performances with ER having, perhaps, a slight advantage at the lower Rsq and SK at the larger Rsq. Under partial heterogeneity (ξ = 0.8), the relative performance of the methods is similar to the one under heterogeneity. The only difference is that RCS-C and TH performs improves somewhat relative to heterogeneity.

## Discussion

Our findings have several implications for the choice of method used to detect association between RVs and binary traits. First, simple logistic regression has good performance when the heterogeneity is not extreme. Second, SKAT and EREC methods using permutations produce similar results and have good performance under all scenarios we tested. However, with permutations, the two methods are very computer intensive. If the computational requirements for SK and ER are problematic, the other three methods we tested (RCS-C, CA and TH) might be useful for selecting “suggestive” genes to be further analyzed using the permutation-based versions of SKAT and EREC. (SKAT with asymptotic assumptions can also be used for selecting suggestive signals, even though, in our experience, it can sometimes become quite conservative - especially for binary traits at lower type I errors and lower sample sizes.) The simple regression (RCS-C) might be useful to select such genes, especially when one does not expect a substantial heterogeneity of RV effects. C-alpha (CA) without permutations (for increased speed) can be used to select promising genes when the relevant covariates explain only a small fraction of the trait variability. The trend and heterogeneity test could be useful for selecting suggestive regions when the heterogeneity of RV effects is expected to be high and the relevant covariates are known to explain a large fraction of the binary trait variability.

## Supporting Information

Table S1 **The conditional distribution of true deleterious classes (Dt ) given PolyPhen predicted classes (Dp) in italics and their marginal distributions (last column and row) in bold.** s is the fitness effect.
(DOC)
