# Supplementary material for: Comparison of Statistical Tests for Association between Rare Variants and Binary Traits
Source: PLoS One. 2012 Aug 9;7(8):e42530. doi: 10.1371/journal.pone.0042530 (PMC3415421; doi:10.1371/journal.pone.0042530)
Supplement: Table S1 — The conditional distribution of true deleterious classes (Dt ) given PolyPhen predicted classes (Dp) in italics and their marginal distributions (last column and row) in bold. s is the fitness effect. (DOC) [file pone.0042530.s001.doc]

Table S1. The conditional distribution of true deleterious classes (D_t_ ) given PolyPhen predicted classes (*D_p_*) in italics and their marginal distributions (last column and row) in bold. s is the fitness effect.

|  | **Benign (*D_p_=0*)** | **Possibly damaging (*D_p_=1*)** | **Probably damaging (*D_p_=2*)** | ***D_t_* marginal distribution** |
| --- | --- | --- | --- | --- |
| **|s|<0.0001 (*D_t_=0*)** | *42.00%* | *14.00%* | *7.40%* | ***26.70%*** |
| **0.0001<|s|<0.001 (*D_t_=1*)** | *9.30%* | *25.50%* | *18.60%* | ***15.70%*** |
| **0.001<|s|<0.01 (*D_t_=2*)** | *40.20%* | *31.30%* | *30.30%* | ***35.60%*** |
| **0.01<|s| (*D_t_=3*)** | *8.40%* | *29.20%* | *46.30%* | ***22.00%*** |
| ***D_p_* marginal distribution** | ***50.80%*** | ***25.70%*** | ***23.50%*** |  |
